# Care and support for women during labour: A review

**DOI:** 10.6026/9732063002001598

**Published:** 2024-11-30

**Authors:** Bama Ramu, Desigamani K., Shankar Shanmugam Rajendran

**Affiliations:** 1 Meenakshi Academy of Higher Education and Research, MAHER (DU) & NPME, College of Nursing, Madras Medical College, Chennai, India; 2 Department of Biochemistry, Meenakshi Medical College Hospital and Research Institute, MAHER (DU), Enathur, Kanchipuram, Tamil Nadu, India; 3 College of Nursing, Madras Medical College, Chennai. (The TN MGR Medical University, Chennai), India

**Keywords:** Childbirth experiences, emotional support, maternal care, midwifery practices, respectful care

## Abstract

The experience of childbirth significantly impacts a woman's mental, emotional and physical well-being requiring unique care varying throughout the process. The World Health Organization (WHO) emphasizes the importance of safe, effective, timely and woman-centred maternal and infant health care. Effective communication, respect and emotional support during labour are critical. However, unclear communication and mistreatment can lead to distress. Nonetheless, empathetic care enhances satisfaction during labour. Labour flexibility also contributes to comfort and control, underscoring the need for holistic, dignified and respectful maternal care. It should be noted that further research is essential to address postnatal care gaps and ensure women's psychological and emotional needs are met globally.

## Background:

Giving birth, a significant event in a woman's life, is both mentally and physiologically [1]. A woman's birth experience can influence her future well-being. The need for support varies during the childbirth process and each woman has unique care requirements [2]. According to the World Health Organization, quality care for women should be safe, effective, timely, efficient and women-centered and it is acknowledged as a vital part of both maternal and infant health [3]. Quality of care is a multidimensional notion influenced by the organization's exterior structure, the environment's administrative aspects and each individual patient's preferences [4]. Birthing mothers need their healthcare providers to integrate clinical knowledge and abilities with interpersonal and cultural competence. Labor and birth are generally classified into phases and stages [5]. For women, it can be an emotional journey to birth instead of definite phases and stages and the postnatal phase is an integral aspect of the laboring experience [6]. Professional support and presence have evolved alongside maternity care. The postnatal time is an underserved area of maternity care and postnatal care recommendations do not address women's basic requirements [7]. Furthermore, a woman's experience with childbirth can differ significantly from that of a caregiver or relative. The person accompanying the woman may focus on more real, observable characteristics while overlooking psychological aspects. It is consequently crucial that women are asked about their experiences [8]. Women have the right to receive dignified, respectful and humane health care during childbirth [9]. Mistreatment of women during childbirth is a grave violation of their fundamental human rights. Such mistreatment can manifest both in interactions with healthcare providers and due to systemic failures at the facility and broader health system levels [10]. Addressing this issue requires developing and applying trustworthy and validated measures to capture women's experiences, ensuring the promotion of respectful, courteous and supportive care.

## Methodology:

**Figure 1:**
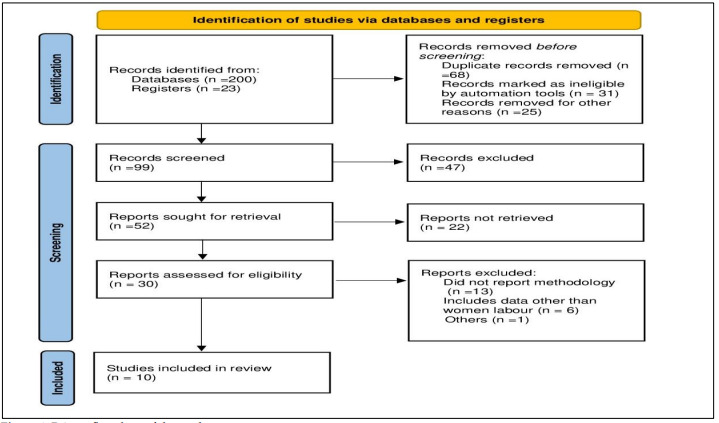
Prisma flowchart of the study

A comprehensive literature search was conducted using Boolean search techniques to identify relevant studies on midwifery, childbirth and maternal care (Figure 1). The databases searched included PubMed, Web of Science and Google Scholar among others. The time frame for the search ranged from 2002 to 2024. The search aimed to capture peer-reviewed articles, reviews, meta-analyses and case reports that focus on midwifery practices, women's birth experiences, maternal care interventions and psychological impacts associated with childbirth.

## Search strategy:

The Boolean search strategy combines keywords and medical subject headings (MeSH) with Boolean operators (AND, OR, NOT) to ensure the inclusion of all relevant studies while excluding irrelevant articles.

## Search terms:

The following keywords were employed during the search: "Midwifery" AND "childbirth" OR "maternal care," "Labor and delivery" OR "birth positions," "Psychological impact" OR "postnatal care," "Women's health" AND "partner perspectives," and "Obstetric interventions" AND "birth outcomes". These keywords were chosen to encompass a wide range of topics related to maternal health, childbirth practices and the experiences of both women and their partners. By combining terms related to medical, psychological and sociocultural aspects of childbirth, the search aimed to capture a comprehensive array of studies relevant to midwifery, labor, delivery positions, postnatal care and obstetric outcomes. Searches were conducted using the databases PubMed, Web of Science and Google Scholar. These platforms were selected for their extensive coverage of peer-reviewed literature and scientific research across various disciplines. PubMed was particularly useful for accessing biomedical and life sciences studies, while Web of Science provided a broad multidisciplinary approach, offering high-quality sources in fields like dentistry, healthcare and biology. Google Scholar was employed to include additional grey literature and interdisciplinary work, further enriching the review with diverse perspectives and research findings.

## Inclusion criteria & exclusion criteria:

The inclusion criteria for the search encompassed studies published between 2002 and 2024, with a focus on peer-reviewed journal articles. Only studies that centered on midwifery, maternal care and labor were considered, including both qualitative and quantitative research. In contrast, the exclusion criteria ruled out articles not available in English, studies addressing unrelated medical conditions and non-research articles such as editorials, commentaries and opinion pieces. This selection process ensured the relevance and rigor of the included studies, concentrating on comprehensive, data-driven research in the field of maternal health.

## Search results:

The initial search retrieved a broad range of articles, which were then screened based on title and abstract. Relevant full-text articles were reviewed and studies were selected for inclusion if they met the predefined criteria. The selected studies reflect a wide array of topics including labor and birth positions, midwifery practices, partner perspectives and maternal healthcare outcomes.

## Review:

The domains of the WHO framework on quality of care for maternal and newborn health can help understand women's delivery experiences. These domains highlight key aspects influencing their perceptions. A significant variation is often observed in how women communicate with healthcare providers during childbirth. The reviewed studies highlight the critical aspects of childbirth experiences, emphasizing the importance of effective communication, respect, emotional support and flexibility in labor practices. Effective communication, as shown by Mackenzie *et al.* (2018) [11] and Isaacs *et al.* (2020) [12], reduces anxiety, improves satisfaction and fosters emotional well-being, while poor communication leads to fear and uncertainty. Respect and dignity, explored by Rosen *et al.* (2004) [13] and Ng *et al.* (2024) [14], significantly enhance maternal mental health, though inconsistent treatment underscores the need for better training in respectful care practices. Continuous and emotional support during labor, as detailed by Williamson *et al.* (2017) [15] and Dawson *et al.* (2018) [16], enhances satisfaction, reduces isolation and improves overall perceptions of labor experiences. Studies by Button *et al.* (2017) [17], Sanders *et al.* (2018) [18] and Shorey *et al.* (2022) [19] emphasize the value of flexibility in labor positions, which improve comfort, reduce pain and empower women. O'Connell *et al.* (2021) [20] found that shared decision-making and effective communication between women, their partners and providers contribute to more positive and collaborative birthing experiences. Collectively, these findings underline the importance of respectful, individualized and supportive care to ensure positive maternal outcomes.

In summary, the literature indicates that women's experiences during intrapartum care are multifaceted, influenced by effective communication, respect, emotional support and the ability to choose their labor positions. Addressing these factors can significantly enhance the quality of care and improve maternal outcomes. A literature review table has been added to Table 1. This systematic review of women's experiences and needs during labor reveals the importance of emotional, physical and informational support in shaping positive birth experiences. Emotional support, such as reassurance and empathy, significantly enhances feelings of safety and reduces anxiety, while its absence can lead to distress and feelings of abandonment. Physical support during labor, including non-pharmacological pain relief measures such as massage and the creation of a calming environment, is highly valued by women as it promotes comfort and a sense of control.

## Discussion:

The findings of this review emphasize the multifaceted nature of care and support needed during labor, particularly for Indian women and align with global standards advocated by the World Health Organization (WHO). Effective communication emerges as a cornerstone of positive childbirth experiences. Studies such as those by Mackenzie *et al.* (2018) [11] and Isaacs *et al.* (2020) [13] demonstrate that clear, compassionate and informative interactions between healthcare providers and women significantly reduce anxiety, foster emotional well-being and improve overall satisfaction. Conversely, ambiguous or dismissive communication often leads to uncertainty and distress, underscoring the need for consistent training and awareness among care providers.

Respectful care is another critical dimension influencing maternal experiences. Rosen *et al.* (2004) [13] and Ng *et al.* (2024) [14] highlight the importance of treating women with dignity during childbirth, revealing a strong association between respectful care and improved maternal mental health outcomes. However, instances of mistreatment indicate systemic gaps that necessitate targeted interventions and accountability within healthcare settings. Emotional and physical support during labour also plays pivotal roles in shaping women's perceptions. Williamson *et al.* (2017) [15] and Dawson *et al.* (2018) [16] underscore that continuous emotional support, including reassurance and encouragement from midwives, reduces feelings of isolation and enhances satisfaction. Furthermore, studies by Button *et al.* (2017) [17], Sanders *et al.* (2018) [18] and Shorey *et al.* (2022) [19] demonstrate that providing women with flexibility in choosing labour positions enhances comfort and reduces pain perception, thereby fostering empowerment and a sense of control. O'Connell *et al.* (2021) [20] further emphasize the value of shared decision-making, where effective communication and collaboration with care providers improve the experiences of both women and their partners.

These findings stress integrating emotional, physical and informational support within maternal care frameworks. Emotional support, such as empathy and reassurance, not only effects of stress and fear. Physical support measures promote comfort and autonomy, including non-pharmacological interventions like massage and personalized birthing environments. Through clear and on-going communication, informational support empowers women to make informed decisions, fostering trust and collaboration with care providers. In the Indian context, where systemic and cultural barriers may further compound challenges in maternal care, it is imperative to prioritize personalized, respectful and evidence-based approaches. Addressing these gaps through improved training, infrastructural support and policy-level interventions can significantly enhance maternal health outcomes and align care practices with global standards of dignity and quality. This comprehensive approach not only fulfils women's physical and emotional needs during labor but also ensures a positive and empowering childbirth experience. The Counseling for Maternal and Newborn Health Care handbook by the World Health Organization (WHO) [21] provides practical guidance for healthcare providers in building effective counseling skills. This resource underscores the value of communication and emotional support in ensuring a positive birthing experience, a component increasingly recognized as essential for both physical and psychological maternal health. Cumpston *et al.* (2019) [22] reinforce the importance of systematic and rigorous approaches in evaluating maternal care practices. Their Cochrane review provides an updated synthesis of the evidence, contributing to the ongoing improvement of maternal healthcare strategies. The methodological rigor of Cochrane reviews ensures the reliability of the evidence, which can be translated into clinical practice to enhance support during labor and childbirth. Similarly, the review by Bohren *et al.* (2017) [23] explores the impact of continuous support for women during labor. Their findings demonstrate that women who receive uninterrupted support are more likely to experience improved outcomes, such as reduced need for interventions like cesarean sections and instrumental deliveries, shorter labor duration and increased satisfaction with the birthing process. The review also highlights the importance of individualized care, which aligns with the recommendations in the WHO handbook [21]. Lunda, *et al.* (2018) highlighted the positive impact of continuous support during labor, including improved maternal satisfaction and reduced intervention rates. Their findings emphasize the need for emotional, physical and informational support, particularly through personalized care from trusted companions. However, challenges in resource allocation and cultural differences must be addressed to ensure all women can access this vital support during childbirth [24].

## Conclusion:

This review highlights the critical role of emotional reassurance, physical comfort measures and clear communication in fostering positive birth experiences and reducing anxiety for women during labor. Effective communication, respect, dignity and flexibility in labor positions significantly enhance maternal satisfaction, providing women with a greater sense of control and comfort during childbirth. On-going improvements in midwifery practices, along with a focus on personalized care, are essential for promoting safer, more supportive and satisfying childbirth experiences, leading to better maternal health outcomes.

## Figures and Tables

**Table 1:** Literature review of the study

**Author(s)**	**Journal Name**	**Objectives**	**Key Findings**
Mackenzie *et al.* (2018) [18]	Midwifery	To investigate the role of communication between midwives and women during labor.	Effective communication reduced anxiety and enhanced satisfaction during labor; women felt more positive about their birth experience when informed and supported.
Isaacs *et al.* (2020) [19]	BMC Psychology	To explore the psychological effects of communication styles used by healthcare providers.	Clear and compassionate communication improved emotional well-being, while ambiguous responses led to fear and uncertainty among Women.
Rosen *et al.* (2004) [15]	Journal of Midwifery & Women's Health	To evaluate women's perceptions of respect and dignity in intrapartum care.	Some women reported positive interactions, while others experienced mistreatment, highlighting the need for improved training in respectful care practices.
Ng *et al.* (2024) [20]	Midwifery	To assess the prevalence of respectful care during childbirth and its impact on maternal outcomes.	Respectful treatment is associated with better maternal mental health outcomes, underscoring the importance of dignity in intrapartum care.
Williamson *et al.* (2017) [17]	BMC Pregnancy and Childbirth	To explore women's experiences of support during labor.	Continuous support from providers enhanced satisfaction and reduced feelings of isolation among women.
Dawson *et al.* (2018) [21]	BMJ Open	To examine the emotional support provided by midwives during labor.	Emotional support from midwives significantly impacted women's perceptions of their labor experience, with many expressing appreciation for encouragement and reassurance.
Button *et al.* (2017) [22]	British Journal of General Practice	To investigate women's preferences and perceptions regarding labor and birth positions.	Women desired more flexibility in choosing birth positions, which they felt could enhance comfort and control during labor.
Sanders *et al.* (2018) [23]	BMC Pregnancy and Childbirth	To explore the implications of birth positions on women's experiences of labor.	Women using various labor positions reported improved comfort and decreased pain perception,
Shorey *et al.* (2022) [24]	Birth	To conduct a meta-synthesis of literature on perceptions of labor and birth positions.	A strong preference for supportive and flexible environments emerged, where women felt empowered to choose their labor positions, positively influencing their overall birth experience.
O'Connell *et al.* (2021) [20]	Women and Birth	To explore the experiences of women and their partners regarding labor and birth.	Both women and partners valued shared decision- making and communication with providers, contributing to a more positive birthing experience.

## References

[1] Downe S (2018). PloS one.

[2] Coates R (2019). Midwifery.

[3] Fair F (2020). PloS One..

[4] Mosadeghrad A.M (2012). Mater Sociomed..

[5] Ängeby K (2024). Eur J Midwifery..

[6] Olza I (2018). BMJ open..

[7] Negron R (2013). Matern Child Health J..

[8] McKelvin G (2021). Women and Birth..

[9] McKnight P (2019). Midwifery..

[10] Bohren M.A (2015). PLoS medicine..

[11] Mackenzie J (2018). Midwifery..

[12] Isaacs N.Z, Andipatin MG. (2020). BMC psychology..

[13] Rosen P (2004). Journal of Midwifery & Women's Health..

[14] Ng Y.X (2024). Midwifery..

[15] Williamson G.R (2017). BMC pregnancy and childbirth..

[16] Dawson A.J (2018). BMJ open..

[17] Button S (2017). British Journal of General Practice..

[18] Sanders R.A (2018). BMC pregnancy and childbirth..

[19] Shorey S (2022). Birth..

[20] O'Connell M.A (2021). Women and Birth..

[21] World Health Organization, Geneva (2013). Counseling for Maternal and Newborn Health Care: A Handbook for Building Skills.

[22] Cumpston M (2019). Cochrane Database Syst Rev..

[23] Bohren M.A (2017). Cochrane Database of Systematic Reviews..

[24] Lunda P (2018). BMC Pregnancy Childbirth..

